# Comparative and evolutionary analyses reveal conservation and divergence of the notch pathway in lophotrochozoa

**DOI:** 10.1038/s41598-021-90800-8

**Published:** 2021-05-31

**Authors:** Xin He, Fucun Wu, Linlin Zhang, Li Li, Guofan Zhang

**Affiliations:** 1 CAS and Shandong Province Key Laboratory of Experimental Marine Biology, Center for Ocean Mega-Science, Institute of Oceanology, Chinese Academy of Sciences, Qingdao, 266071 China; 2 Laboratory for Marine Biology and Biotechnology, Pilot National Laboratory for Marine Science and Technology, Qingdao, 266237 China; 3 University of Chinese Academy of Sciences, Beijing, 100039 China; 4 Laboratory for Marine Fisheries Science and Food Production Processes, Pilot National Laboratory for Marine Science and Technology, Qingdao, 266237 China; 5 National and Local Joint Engineering Laboratory of Ecological Mariculture, Qingdao, 266071 China; 6 The Innovation of Seed Design, Chinese Academy of Sciences, Wuhan, 430072 China

**Keywords:** Molecular biology, Evolution

## Abstract

Lophotrochozoan species exhibit wide morphological diversity; however, the molecular basis underlying this diversity remains unclear. Here, we explored the evolution of Notch pathway genes across 37 metazoan species via phylogenetic and molecular evolutionary studies with emphasis on the lophotrochozoans. We displayed the components of Notch pathway in metazoans and found that *Delta* and *Hes/Hey*-related genes, as well as their functional domains, are duplicated in lophotrochozoans. Comparative transcriptomics analyses allow us to pinpoint sequence divergence of multigene families in the Notch signalling pathway. We identified the duplication mechanism of a mollusc-specific gene, *Delta2*, and found it displayed complementary expression throughout development. Furthermore, we found the functional diversification not only in expanded genes in the Notch pathway (*Delta* and *Hes/Hey*-related genes), but also in evolutionary conservative genes (*Notch*, *Presenilin*, and *Su(H)*). Together, this comprehensive study demonstrates conservation and divergence within the Notch pathway, reveals evolutionary relationships among metazoans, and provides evidence for the occurrence of developmental diversity in lophotrochozoans, as well as a basis for future gene function studies.

## Introduction

Lophotrochozoa is a monophyletic group of animals that includes Platyhelminthes, bryozoans, brachiopods, annelids, molluscs, and other animals that share a common ancestor. Lophotrochozoan species exhibit a high level of biodiversity; for example, Mollusca is one of the richest groups of animals containing over 100,000 different species^1^, and over 16,500 species of Annelida have been described worldwide^2^. Therefore, the superphylum Lophotrochozoa is essential to our understanding of metazoan evolution.

Early developmental pathways have been shown to play a vital role in the evolution of biodiversity, such as pathways that control embryonic development involving transforming growth factor β (TGF-β), wingless/integrated (Wnt), receptor protein tyrosine kinase (RPTK), Janus kinase/signal transducer and activator of tran-ions (Jak/STAT), Hedgehog, retinoic acid signalling (RA), hox cluster (Hox), and Notch^3^. In particular, the Notch pathway is essential for regulating cellular identity, proliferation, differentiation, and apoptosis via lateral inhibition, lineage decisions, and boundary induction, which all play vital roles in metazoan development^4,5^.

**Figure 1:**
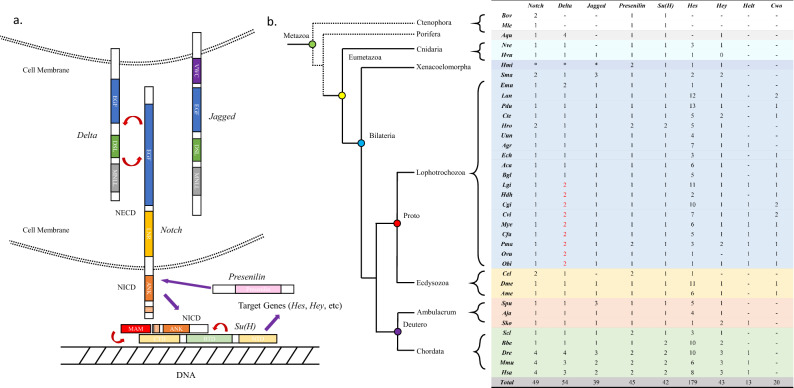
Major genes of the Notch pathway. (**a**) Diagram of the Notch pathway. (**b**) Species colours represent different phyla. Dashed lines represent unresolved phylogenetic positions for ctenophores and sponges. Lines indicate none and “*” indicates lack of a proteome. Species abbreviations are described in the “Methods” section.

The Notch pathway contains the *Notch* receptor, the ligands *Delta* and *Jagged* (known as *Serrate* in *Drosophila melanogaster*), the regulatory factors *Fringe*, *Numb*, *Deltex*, *Mastermind*, *Presenilin,* and *Nrarp*, the transcription factor CSL family (invertebrate *Suppressor of Hairless* (*Su(H*)), vertebrate *CBF1* (also known as *rbpj*), and nematode *LAG-1*; *Su(H)* is used in this paper), and the *Hes* (*Hairy/enhancer of Split*)*/Hey* (*Hairy/Enhancer of Split related with YRPW motif*) target gene family^5^. The *Notch* gene can be divided into three parts: an extracellular domain (NECD) consisting of 29–36 epidermal growth factor (EGF) repeats and three Lin12/Notch (LNR) repeats, a transmembrane (TM) domain, and an intracellular domain (NICD) that includes several ankyrin (ANK) repeats and a region containing proline, glutamate, serine, and threonine (PEST)^5^. The *Notch* ligand *Delta* contains a Delta/Serrate/Lag (DSL) domain, which is crucial for its interactions with the *Notch* receptor, whereas its extracellular regions contain several EGF repeats. Some genes that have lost EGF repeats or other motifs are defined as *Delta-like* (*Dll*). *Jagged,* another *Notch* ligand, contains a von Willebrand factor type C (VWC) domain in addition to the domains in *Delta*. The vital transcription factor *Su(H)* has the following functional domains: C-terminal domain (CTD), beta–trefoil domain (BTD), and the N-terminal domain (NTD). Both the BTD and NTD contact DNA, and the BTD and CTD interact with NICD, whereby the CTD binds both the ANK of NICD and *Mastermind* (MAM)^6–8^. *Presenilin* encodes a multi-span membrane protein and catalytic subunit that only contains a Presenilin domain^5^. The terminal genes of the Notch pathway include *Hes*/*Hey*-related genes that belong to group E of the basic helix-loop-helix (bHLH) superfamily (including *Hes*, *Hey*, *Helt* (also known as *Hesl*)*,* and *Clockwork orange* (*Cwo*)) and encode a bHLH domain as well as a hairy/orange domain^9^. The pathway is exhibited in Fig. 1a.

Previous studies have indicated that the Notch pathway is conserved in metazoans^10^, and traditional model organisms have revealed the various functions of the Notch pathway, which includes regulation of polarity because its loss results in abnormal anterior–posterior polarity or incorrect left–right asymmetry in somites^11,12^. The Notch signalling pathway also plays central roles in vertebrate somitogenesis^13–15^ and differentiation of the epidermis and cilia^16^. Consequently, the function of the Notch pathway in non-model organisms has received considerable attention. In the sponge *Amphimedon queenslandica,* the Notch pathway is involved in regulation of diverse cell types during development^17^, and it plays essential roles during nervous system development^18^ and boundary formation^19^ in cnidarians. Recent lophotrochozoan studies have associated the Notch pathway with formation of germ layers, neurogenesis, segments, and chaetogenesis^20–24^; however, it remains unclear how the pathway evolved in these organisms.

The comparative genomic and transcriptomic analysis was widely used in evolutionary developmental biology^25,26^. Here, we investigated the conservation and divergence of Notch pathway gene components and annotated genes encoding molecules that affect early development of 37 metazoan species. In addition, we performed a comparative genomic study on core components of the Notch pathway (*Notch*, *Delta*, *Jagged*, *Presenilin*, *Su(H)*, *Hes*, *Hey*, *Helt*, and *Cwo*) in metazoans and elucidated their evolutionary relationships using phylogenetic analysis. Consequently, this study clarified the molecular mechanisms responsible for evolution of the novel molluscan gene *Delta2* and elucidated patterns of Notch pathway gene expression using comparative transcriptome during development, thus providing a basis for further evo-devo research on lophotrochozoans.

## Methods

### Data collection and Notch pathway gene identification

We selected 37 metazoan species for our study: two Ctenophora, *Beroe ovata* (Bvo; version 1.0) and *Mnemiopsis leidyi* (Mle); one Porifera, *Amphimedon queenslandica* (Aqu; version 1.0); two Cnidaria, *Hydra vulgaris* (Hvu; version 1.0) and *Nematostella vectensis* (Nve; version 1.0); one Acoela, *Hofstenia miamia* (Hmi; version 1.0); 20 Lophotrochozoa, including one Brachiopoda *Lingula anatina* (Lan; version 2.0), two Platyhelminthes, *Schistosoma mansoni* (Sma; version 2) and *Echinococcus multilocularis* (Emu; version EMULTI002), four Annelida, *Platynereis dumerilii* (Pdu), *Helobdella robusta* (Hro; version 1.0), *Urechis unicinctus* (Uun) and *Capitella teleta* (Cte; version 1.0), thirteen Mollusca, *Acanthopleura granulate* (Agr), *Aplysia californica* (Aca; version 3.0), *Elysia chlorotica* (Ech; version 2.0), *Biomphalaria glabrata* (Bgl; version 1.0), *Lottia gigantea* (Lgi; version 1.0), *Haliotis discus hannai* (Hdh), *Crassostrea gigas* (Cgi; version oyster_v9), *Crassostrea virginica* (Cvi; version 3.0), *Mizuhopecten yessoensis* (Mye; version 2.0), *Chlamys farreri* (Cfa), *Pinctada fucata martensii* (Pma), *Octopus bimaculoides* (Obi; version 2.0), and *Octopus vulgaris* (Ovu; version 1.0); one Nematoda, *Caenorhabditis elegans* (Cel; version WBcel235); two Arthropoda, *Drosophila melanogaster* (Dme; version Release 6 plus ISO1 MT) and *Apis mellifera* (Ame; version 3.1); two Echinodermata, *Strongylocentrotus purpuratus* (Spu; version 5.0) and *Apostichopus japonicus* (Aja, version 1.0); one Hemichordata, *Saccoglossus kowalevskii* (Sko; version 1.1); and five Chordata, *Styela clava* (Scl, version 2.0), *Branchiostoma belcheri* (Bbe; version Haploidv18h27), *Danio rerio* (Dre; version GRCz11), *Mus musculus* (Mmu; version GRCm39), and *Homo sapiens* (Has; GRCh38.p13). All genomes and proteomes were downloaded from the National Center for Biotechnology Information (NCBI) database (https://www.ncbi.nlm.nih.gov/), except those of *P. fucata martensii*, which was obtained from GigaDB (http://gigadb.org/dataset/100240)^27^, *C. farreri*, which was obtained from CfBase (http://mgb.ouc.edu.cn/cfbase/html/)^28^, and *A. granulata*, which was obtained from https://alabama.app.box.com/s/1hsryfff61i01qrljyasrjnu8j7qg2nj. The genomes of *M. leidyi*, *P. dumerilii*, *U. unicinctus*, and *H. discus hannai* have not been published yet. The genome and protein sequence datasets were searched for each species. Genes were identified using the default protein-to-nucleotide Basic Local Alignment Search Tool (tblastn) in NCBI. Sequences with the lowest E-value were selected for analysis after all matches with unexpected domain architecture had been discarded after being corrected by Gene Wise^29^.

We defined *Notch* as a gene containing several EGF domains, three LNR repeats, a TM domain, and several ANK repeats, *Delta* as a gene containing a DSL domain, an MNLL domain, and EGF domains, and *Jagged* as a gene having the same domains as *Delta*, with an additional VWC domain. Similarly, Presenilin and BTD/LAG1-DNAbind domains belonged to *Presenilin* and *Su(H)*, respectively. The bHLH domains of *H. sapiens* and *D. melanogaster* were used as query sequences in tblastn searches for *Hes/Hey*-related (*Hey*, *Hes*, *Helt,* and *Clockwork*) members in metazoans.

HMMER3.3 (http://hmmer.org/) was used to screen significant domains in Notch pathway genes with a low cut-off threshold value of E-5 against all datasets. Profile hidden Markov models (profile HMMs) were accessed via Pfam (http://pfam.xfam.org/). All protein domain visualisations were checked by scanning sequences using the NCBI Conserved Domain search^30^ and Simple Modular Architecture Research Tool (SMART)^31^. Pfam accession numbers are available in Supplementary Table S1.

### Phylogenetic analysis

We constructed phylogenetic trees using 27 species (*A. queenslandica* and *H. vulgaris* as outgroups) whose Notch pathway protein sequences were well assembled. Multiple alignments were produced using Clustal W in MEGA X software under default parameters that were manually adjusted^32^. TrimAL (http://phylemon2.bioinfo.cipf.es/) was used to trim protein sequences under automated1 mode. Since the phylogenetic trees constructed using Bayesian Inference (BI), neighbour-joining (NJ), and maximum likelihood (ML) methods were consistent, only trees of nine core components constructed using the ML method with the best fit model (WAG + CAT) in Fasttree (version 2.1.11, 1000 bootstrap replicates) are shown^33^. Trees were prepared using FigTree (version 1.4.3), iTOL (https://itol.embl.de/), and EvolView (https://evolgenius.info//evolview-v2/#login). All BLAST gene query sequences were obtained from published papers^34,35^.

The phylogenetic trees of nine core genes (*Notch*, *Delta*, *Jagged*, *Presenilin*, *Su(H)*, *Hes*, *Hey*, *Helt*, and *Cwo*) were constructed using entire protein sequences (IDs listed in Supplementary Tables S2 and S3). The DSL family tree was constructed using the DSL domain of each gene. If a gene contained more than one DSL domain, ‘-’ was used to denote their order in the gene.

### Transcriptomic analysis of gene expression

Gene expression levels were measured as reads per kilobase per million (RPKM) or fragments per kilobase million (FPKM). Transcriptomic data (RPKM) from the developmental stages and adult tissues of *C. gigas* were obtained from NCBI (accession GSE31012) and the supplementary material of a published paper^36^. Transcriptomic data (RPKM) for *P. martensii* were obtained from GigaDB and the supplementary material of the associated publication^27^. The raw data of *A. queenslandica*, *A. japonicus*, *H. discus hannai*, *M. yessoensis*, *M. leidyi*, *S. clava*, and *U. unicinctus* were obtained from the study by Wang et al.^25^. The RNA sequencing values for *D. melanogaster* were retrieved from the FlyBase website (http://flybase.org/). The time-course of *D. rerio* was derived from a previous report^37^. For species with no reference genomes, the sratoolkit (version 2.10.8) was used to convert SRR raw data downloaded from NCBI to FASTq format. Transcriptome assembly was performed using Trinity (version 2.2.0) and RSEM (version 1.3.3), which were used to calculate the expression profiles. For species with completely spliced genomes, Hisat2 (version 2.1.0) was used to build index and mapping, SamTools (version 1.11) to format conversion, and Cufflinks (version 2.2.1) to calculate the expression levels. Data were visualised using TBtools (version 1.089)^38^*.* Owing to differences in measurement methods of gene expression levels, only expression trends between species were analysed. Abbreviation definitions and divisions of developmental stages are available in Supplementary Table S4.

## Results

### Identification of Notch pathway genes and related domains

To investigate the diversity of development-related domains in different species, we selected 18 key domains from developmental pathways (including TGF-β, Wnt, Jak/STAT, RPTK, Notch, Hedgehog, RA, Fox, Hox, and ERK) and predicted the number of domains across 34 species whose genomes had been completely sequenced. The EGF, Homeobox, bHLH, and SH2 domains were extensively expanded across metazoans (16,894, 4300, 2259, and 1966, respectively; Table S5), whereas genes encoding ERK-JNK_inhib, TALPID3, HH_signal, RAI16-like, and STAT_bind were relatively conserved (33, 41, 72, 74, and 76, respectively). Overall, EGF was the most expanded domain in lophotrochozoans, and DSL and bHLH also showed high numbers of domains (highlighted in red). Notably, genes encoding the EGF, DSL, and bHLH domains were all related to the Notch pathway. To elucidate the evolution of diversity in Notch pathway components, we identified nine functional domains that serve essential roles in the Notch pathway (Table S6). With the exception of EGF, DSL, and bHLH domain expansions, we found that the Presenilin and BTD/LAG1-DNAbind domains only existed in *Presenilin* and *Su(H)*, respectively, indicating they were conserved.

To further investigate the origin and evolution of the Notch pathway, we examined the genomes of 37 metazoan species of different evolutionary status, with an emphasis on 20 lophotrochozoans including Platyhelminthes, Brachiopoda, Annelida, and Mollusca. We annotated and compared the numbers of core Notch pathway genes, including the *Notch* receptor, the *Delta* and *Jagged* ligands, the γ-secretase complex component *Presenilin*, the transcription factor *Su(H)*, and *Hes/Hey*-related target genes (Fig. 1b). *Notch*, *Presenilin,* and *Su(H)* were identified in the ancestral metazoans Ctenophora (*B. ovata* and *M. leidyi*) and Porifera (*A. queenslandica*), indicating that major Notch pathway components were present before metazoan ancestors; however, neither *Delta* nor *Jagged* were identified in Ctenophora. *Hes/Hey*-related genes were found in Cnidaria, whereas *Helt* and *Cwo* first appeared in Lophotrochozoa. Notably, *Delta* was duplicated in several Mollusca including bivalves, gastropods, and cephalopods (Fig. 1b, red). The ancestral mollusc Polyplacophora *A. granulata* had *Helt*, indicating that some gastropods probably lost this gene, such as *A. californica*, *E. chlorotic*, *H. discus hannai*, and *B. glabrata*. Notch pathway genes also showed expansion in vertebrates resulting from two genome-wide duplications (2R), which provided genetic variation during vertebrate evolution^39,40^ However, it remains unclear how *Delta* was duplicated in molluscs.

### DSL gene family evolution

**Figure 2:**
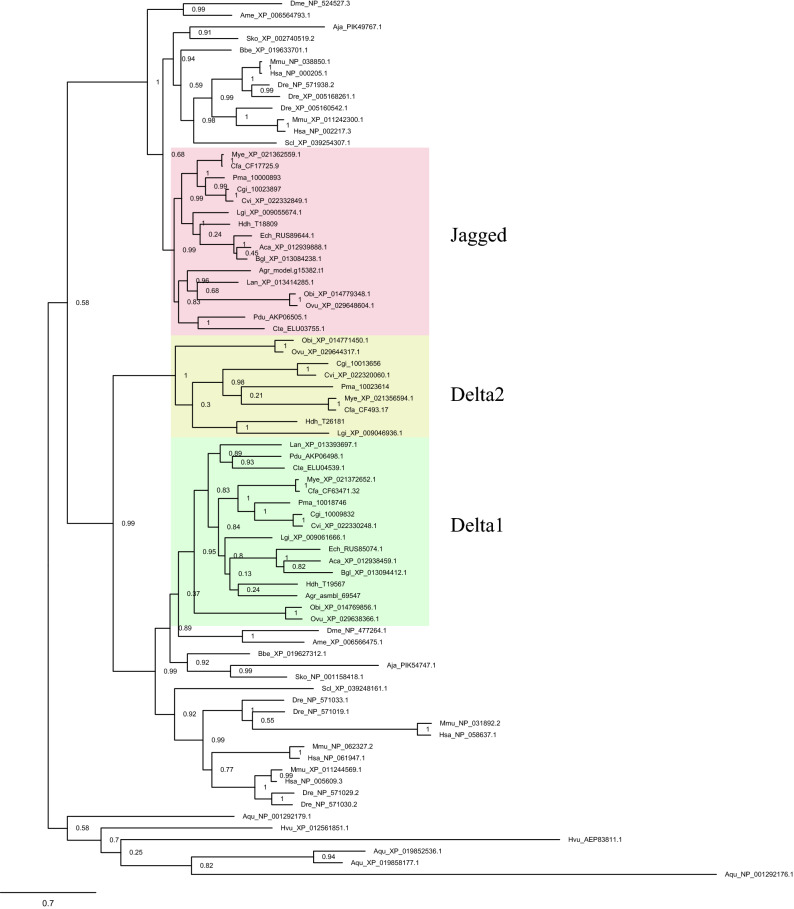
Phylogeny of *Notch* ligands. Phylogenetic tree of *Delta* and *Jagged* genes in metazoans, as determined using the maximum likelihood method. The tree was constructed using 76 ligand genes from 27 complete metazoan genomes (including 16 lophotrochozoans; Platyhelminthes was not included owing to their specific parasitic lifestyle). Green indicates *Delta1* in Lophotrochozoans. Yellow indicates *Delta2.* Pink indicates *Jagged* (*Serrate* in *D. melanogaster*) in Lophotrochozoans. Fasttree support values are shown at the basal node. Sequence IDs are shown in Supplementary Table S2. The *Delta* protein sequences are shown in Supplementary Data 1.

The Notch pathway ligands *Delta* and *Jagged* have similar domain architectures (MNLL, DSL, and EGF) and both belong to the DSL family. Phylogenetic analysis indicated that *Delta* and *Jagged* descended from the same ancestor in early metazoans, such as the sponge *A. queenslandica* and the hydra *H. vulgaris*, which concurred with the results of previous studies^34^ (Fig. 2). In addition, *Delta* displayed a broadly similar topology to *Jagged* in eumetazoans, yet *Jagged* appeared later than *Delta*. Surprisingly, we found that molluscs, including bivalves, cephalopods, and gastropods, fell into another *Delta* clade, which we named *Delta2* (Fig. 2). Interestingly, *Delta2* occurred both independently and appeared later than *Delta1*, indicating functional gene differentiation. Phylogenetic analyses revealed that *Delta2* was absent in the ancestral mollusc Polyplacophora *A. granulata* but was present in other molluscs, indicating that *Delta2* originated in the ancestor of Gastropoda, Cephalopoda, and Bivalvia. Moreover, *Delta2* was identified in *L. gigantea* and *H. hannai* but lost in gastropods *B. glabrata*, *E. chlorotica,* and *A. californica*.

**Figure 3:**
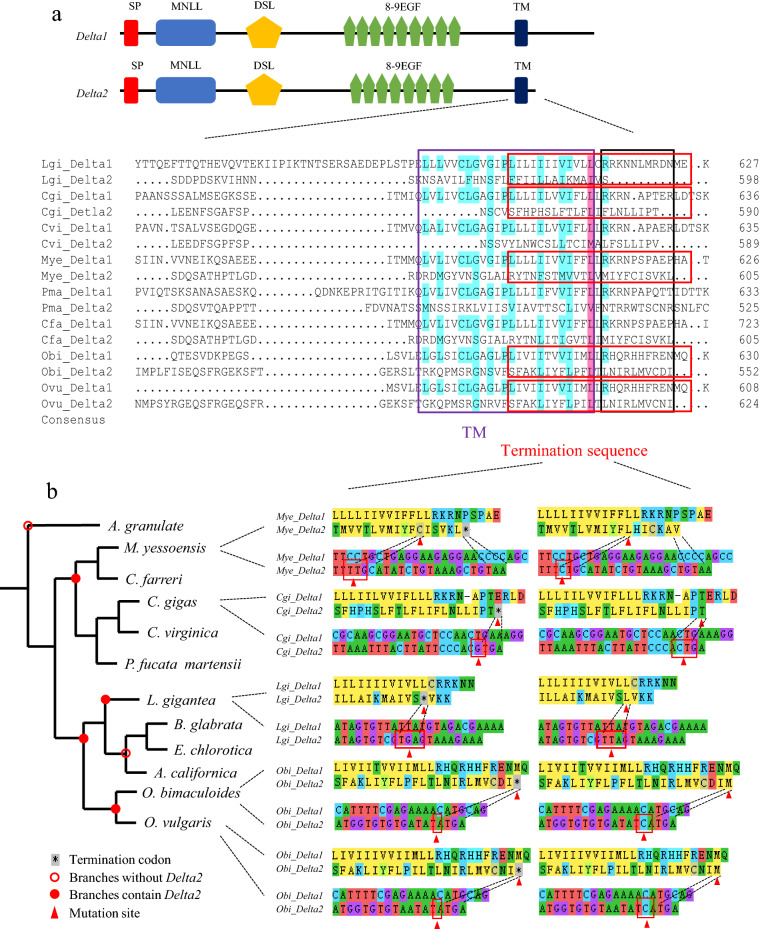
Early termination mechanisms in mollusc-specific *Delta2*. (**a**) *Delta1* and *Delta2* gene structures and blast (DNAMAN) results. The protein sequences indicated in the purple frame are TM domains whereas those indicated in the black frame are common termination positions for *Delta2*. The sequences in the red frame were analysed in detail in (**b**). (**b**) *Delta2* termination mechanisms in different molluscs. Red solid circles indicate branches containing *Delta2.* Red hollow circles indicate no *Delta2*. Both amino acid and nucleotide sequences are displayed here, and the left column includes the termination sequences in lophotrochozoan species whereas the right column denotes sequences after correction. The colours of amino acids represent the functions of codons; amino acids of the same colour have similar functions. The same colour nucleotide represents the same base. The amino acids with a dashed line corresponding to bases in the red frames and triangles represent mutation codon sites in *Delta2*. “*” means termination codon. MEGA X was used for visualizing mutation sites.

Next, we analysed the mechanism of molecular evolution underlying *Delta* duplication. Although domain architecture was quite well conserved between *Delta1* and *Delta2*, the *Delta2* sequences were shorter than those of *Delta1* (Fig. 3a). Notably, we found that the early termination in *Delta2* that occurred approximately 1–10 amino acids after the arginine (R) of *Delta1* downstream may have caused this difference (Fig. 3a, black frame). The terminal patterns are clarified in Fig. 3b. A G base was inserted in the termination sequences of *Delta2* of the bivalve *C. gigas,* which caused the terminal TGA codon and resulted in early termination. In the Gastropoda *L. gigantea,* early termination occurred owing to a mutation in the second leucine (L) codon (red triangle) in the termination sequences, resulting in the termination codon TTA being corrected to TGA. In *M. yessoensis*, the C base was deleted before cysteine (C, red triangle) in termination sequences, which led to a frameshift mutation. Similarly, deletion of the second C base in isoleucine (I) in Cephalopoda *O. bimaculoides* and *O. vulgaris* resulted in a frameshift mutation in *Delta2*, likely leads to early termination (Fig. 3b).

**Figure 4:**
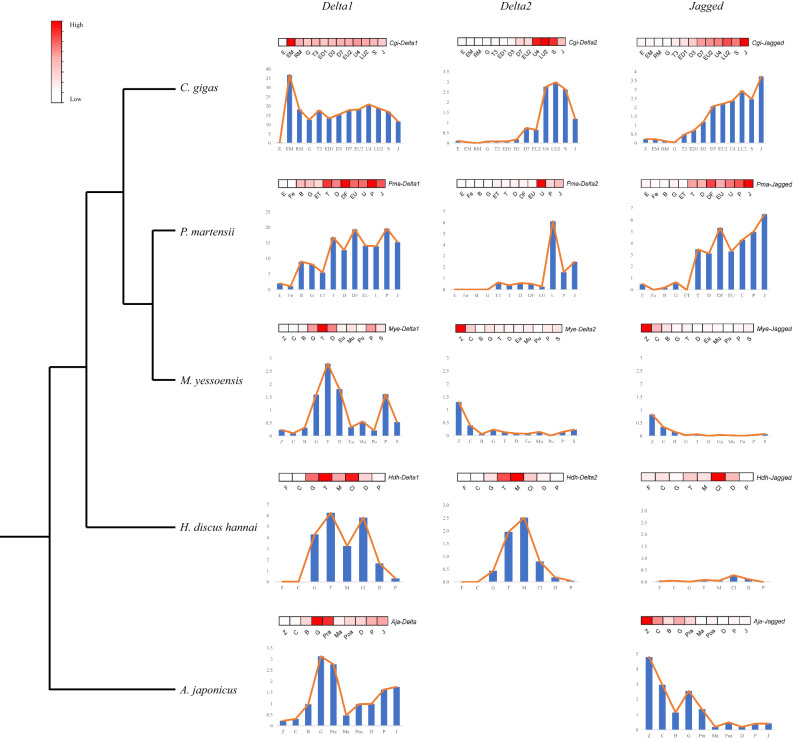
Expression pattern of *Notch* ligands. Transcriptomic data of five species throughout development (*C. gigas*, *P. martensii*, *M. yessoensis*, *H. discus hannai*, and *A. japonicus*). Heatmaps in red display the expression trends of genes. The X-axis of histogram shows the developmental points and the Y-axis denotes fragments per kilobase million (FPKM) or reads per kilobase per million (RPKM). Sequence IDs are shown in Supplementary Table S2. Developmental point and species name abbreviations are described in Supplementary Table S4.

To further investigate the functional differentiation between the ligands in Mollusca, we analysed expression patterns throughout development using transcriptomic data of Mollusca *C. gigas*, *P. martensii*, *M. yessoensis*, *H. discus hannai*, and Echinodermata *A. japonicus* (outgroup) (Fig. 4). The figure shows that *Delta* or *Delta1* were generally highly expressed during the blastula and early larva stages, and *Delta* genes displayed complementary expression throughout development in Mollusca. In *M. yessoensis*, *Delta1* was increased during the gastrula (G), trochophore (T), and D-shaped larva (D) stages, whereas *Delta2* was highly expressed during the zygote (Z) and 2–8 cell (C) stages. In *C. gigas* and *P. martensii*, *Delta2* was increased after the umbone larva stage, unlike *Delta1*. In *H. discus hannai*, *Delta2* was upregulated in the middle veliger stage (M), whereas *Delta1* showed downregulation at this stage. It seemed that *Delta2* was a negatively regulated gene of *Delta1*. Moreover, in *C. gigas* and *M. yessoensis*, the expression pattern of *Jagged* was more similar to *Delta2* than *Delta1*, whereas in *P. martensii* and *H. discus hannai* the expression pattern of *Jagged* was more similar to *Delta1*. Intriguingly, the expression level of *Delta1* was generally higher than that of *Delta2* (Fig. 4; Table. S4). This might result from the loss of the TM domain in *Delta2*, which results in failure of the downstream signal transmission. We also noticed that the expression level of *Mye-Delta2*, *Mye-Jagged*, and *Hdh-Jagged* was very low. As discussed above, the different expression patterns suggest functional divergence between *Delta1* and other ligands after duplication. The ligands of the Notch pathway may coordinate with each other and work together.

To explore how DSL expanded, we screened 138 DSL family genes in lophotrochozoan species and constructed an unrooted phylogenetic tree (Fig. S1a). All DSL family genes were divided into four groups (Fig. S1b) and split the tree into two branches: one branch contained genes encoding DSL or DSL tandem repeats but not the EGF domain (purple clades), being present only in bivalves, whereas the other branch contained genes encoding the DSL, EGF, and other domains (blue clades), including *Delta* and *Jagged* clades. The tree shows that the expansion of DSL mainly resulted from tandem repeats present among bivalves, and the branch length show that DSL combined with EGF underwent rapid differentiation.

### *Hes/Hey*-related gene family evolution

**Figure 5:**
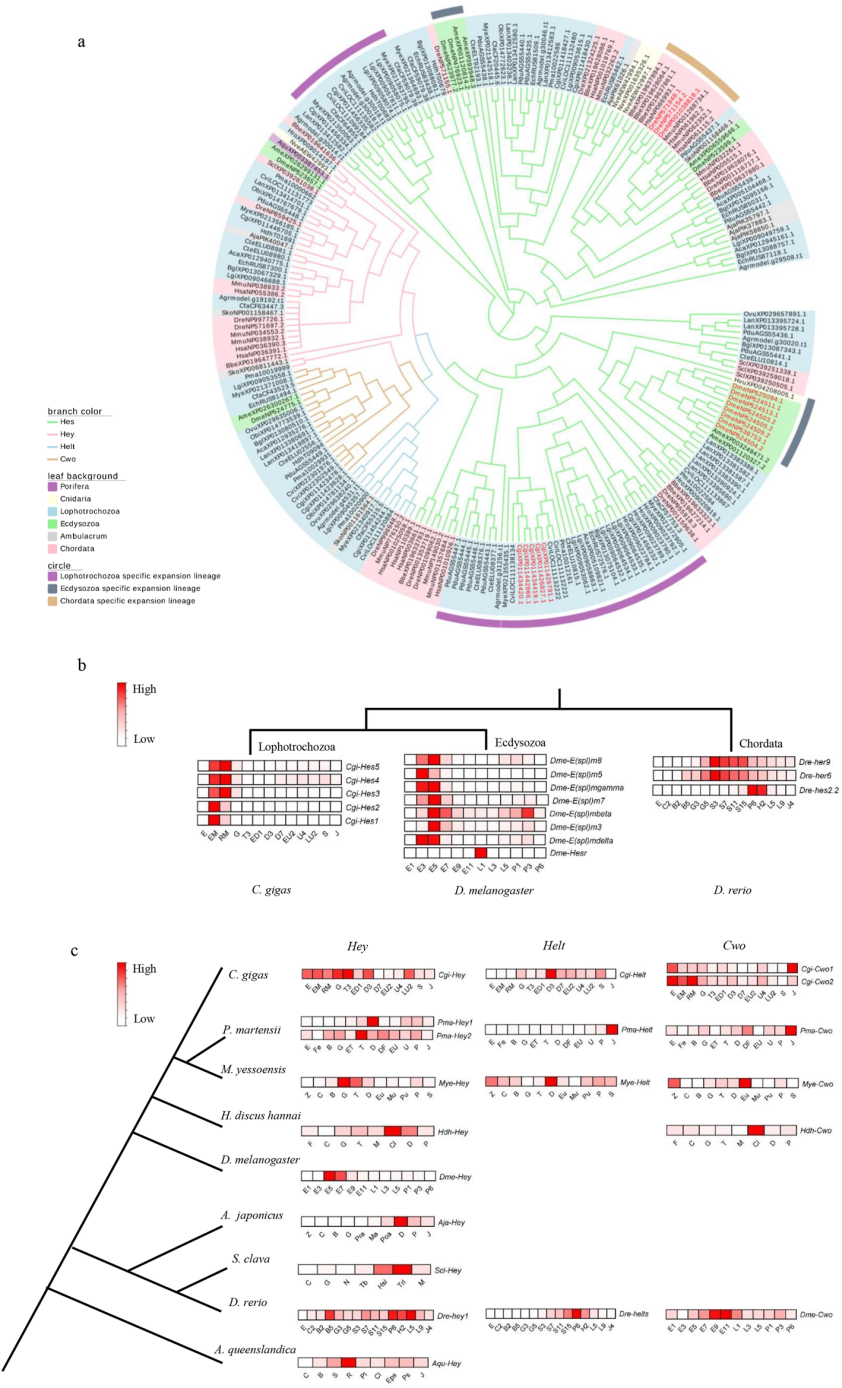
Phylogeny and transcriptome of *Hes/Hey*-related gene family across metazoans. (**a**) A phylogenetic tree was constructed by aligning the bHLH domain of 233 genes. Red labels indicate expanded *Hes* gene clusters that are analysed in (**b**). (**b**) Transcriptomic data of expanded *Hes* gene clusters in lophotrochozoan, Ecdysozoa, and Chordata. (**c**) Transcriptomic data of *Hey*, *Helt*, and *Cwo* in nine species. Sequence IDs are shown in Supplementary Table S3. bHLH domain protein sequences are given in Supplementary Data S3. Developmental point and species name abbreviations are described in Supplementary Table S4.

To investigate divergence within terminal Notch pathway genes, we constructed an unrooted molecular phylogenetic tree of *Hes/Hey*-related family members (*Hes*, *Hey*, *Helt*, and *Cwo*) using the bHLH domain (Fig. 5a). *Hes*, *Hey*, *Helt,* and *Cwo* fell into different clades, whereas *Hes* was expanded in some species lineages including lophotrochozoans, ecdysozoans, and chordates (genes in red), which was also demonstrated in previous studies^35^. We selected several species from different expanded lineages of *Hes* genes for comparative transcriptome analysis (Fig. 5b). The *Hes* gene clusters of the Lophotrochozoa *C. gigas* and the Ecdysozoa *D. melanogaster* were expressed almost during the embryo development stages, implying conserved and specified functions of *Hes* clusters in expanded lineages. However, in Chordata *D. rerio*, *Hes* genes were significantly upregulated during the segmentation, pharyngula, and hatching stages, which displayed that, although the expression pattern of expanded genes of one species was similar, the expression differences and functional differentiations of expanded genes among different specific expanded lineages existed as well. *Hey* was conserved, whereas *Helt* and *Cwo* were novel in the *Hes*/*Hey*-related family, and they displayed different expression patterns not only in lophotrochozoans but also in other species (Fig. 5c). These results suggest that the terminal genes of the Notch pathway play different roles in different lineages, which could be responsible for the phenotypic differentiation in larval stages among species.

### *Notch, Presenilin*, and *Su(H)* evolution

The evolutionary relationships of *Notch*, *Presenilin*, and *Su(H)* in Lophotrochozoa from Porifera to Chordata were clarified by phylogenetic analysis. In lophotrochozoans, *L. anatine* showed a closer relationship with *P. dumerilii* and *C. teleta* than with molluscs in phylogenetic trees of *Notch* and *Presenilin* (Figs. S2 and S3); however, these relationships differed for *Su(H)* (Fig. S4). We then analysed gene developmental expression profiles of the three genes or gene families in 11 species for which comprehensive developmental transcriptomic data are available. Transcriptomic analysis revealed that *Notch*, *Presenilin,* and *Su(H)* were highly expressed during the early embryo developmental stages of most species, but we also found lineage-specific developmental expression patterns of these genes in *A. queenslandica* and *U. unicinctus*, which indicates gene expression divergence of these conserved genes in a clade-specific mode (Fig. S5).

## Discussion

The Notch pathway has been well studied in model animals such as *D. melanogaster*, *C. elegans*, and vertebrates^4^; however, few studies have investigated the Notch pathway in lophotrochozoans. This study significantly expands our knowledge of conservation and divergence within Notch pathway multigene families and elucidates the evolution of core Notch pathway genes in metazoans, with a strong focus on lophotrochozoan species. Interestingly, we found a novel *Delta2* gene in molluscs and proposed the molecular mechanisms of its evolution. Moreover, comparative omics-based analyses revealed differences in gene structure and function between species, whereas gene expression patterns were also observed throughout development, particularly for *Hes*/*Hey*-related genes. Together, our findings enrich our understanding of the Notch pathway and provide a powerful approach for exploring the evolution of developmental pathway genes.

In this study, we found that Notch pathway gene domains could be traced back to Ctenophora, considered one of the earliest evolving extant species^41^ (Fig. 1b). Previous studies only verified the Notch pathway genes in Porifera^17^, but it is still uncertain whether Notch pathway genes are functional in Ctenophora^4^. A recent study determined that some Notch pathway domains, such as MNLL and DSL, existed not only in animals, but also in choanoflagellate species, suggesting that *Notch* and *Delta* appeared much earlier than previously thought^42^. The expansion of DSL and EGF domains in lophotrochozoans has also been associated with the recombination of Notch ligands and some are considered to be *Delta-like* genes^20^ (Fig. S1a), indicating that the functional evolution of ligand genes is probably ongoing, which was consistent with the varying expression patterns of Notch ligands observed in the current study^43,44^. We also found complementary expression patterns between *Delta1* and *Delta2* in Mollusca (Fig. 4); one of the possible reasons is both genes shared MNLL and DSL domains and EGF repeats, the functions of which are likely complementary, interchangeable, or antagonistic^45,46^.

Interestingly, we identified the appearance of *Delta*2 in Mollusca that may have resulted from early termination near arginine after *Delta* duplication (Fig. 3) and we speculated the reasons for early termination differed between species probably owing to high selection pressure and long differentiation time, leading to large sequence variation. In general, duplication of evolutionary genes could lead to non-, sub-, neo-, and synfunctionalisation^47,48^. Because of the different expression pattern (Fig. 4), *Delta* duplication in molluscs likely resulted in neofunctionalisation, meaning that *Delta1* retained its ancestral function and *Delta2* acquired new functions. In *C. gigas* and *P. martensii, Delta2* expression was notably upregulated during umbo larva (Fig. 4). As is known, shells, gills, feet, and eye spots are formed at the umbo larva stage^36^, and thus the observed expression pattern is likely related to specific characteristics that are crucial for phenotype differentiation^21,34^. Indeed, recent studies have revealed that the Notch pathway plays a key role in shell colour, which is crucial for measuring economic value^20,49^.

The terminal genes exhibited gene expansion and expression differentiation among metazoans in this study (Fig. 5). The expanded genes, which probably resulted from gene duplication^35^, showed similar expression patterns associated with embryonic development among *C. gigas* and *D. melanogaster*, but the expression pattern was different from that of *D. rerio*. Unlike the *Hes* genes clusters (also called *E(spl)* genes) in *D. melanogaster*, which had an upregulated expression during the stages of blastoderm and gastrula^50^, the duplicated terminal genes expressed in *D. rerio* were consistently highly expressed after the gastrula and segmentation stages (Fig. 5b), likely resulting from periodic activation of the Notch pathway to control somitogenesis^51,52^. The functional differentiation of other *Hes/Hey*-related genes were also reported in previous studies. For example, Gazave et al*.* found that *Hes* participates in chaetal sac formation in *P. dumerilii*, whereas Rivera et al. found that *Hes* affects the segmentation process in *H. robusta*^23,34^. In mammals, *Hes* controls cellular differentiation and leads to neuronal development abnormalities^53^, whereas the novel gene *Helt* (*Hesl*) is reportedly regulated by *Notch* and plays essential roles in neuronal differentiation^54,55^; however, the functions of these genes in lophotrochozoans remain unclear. Although *Cwo* was found to be a transcriptional repressor and novel circadian pacemaker component in *D. melanogaster*^56^, it is uncertain whether the *Cwo* and Notch pathways are closely connected.

*Notch*, *Presenilin*, and *Su(H)* are conserved genes in the Notch pathway, and displayed high expression levels during the early developmental stages of most species (Fig. S5). Although the expression patterns of these genes in species were similar, the functions were diverse as well. It was reported that, during the embryo stage of Cnidarian *N. vectensis*, *Notch* broadly crossed several tissues including the pharyngeal, body wall endodermal, and ectoderm^18^, as well as in another Cnidarian *H. vulgaris*^57^. In Mollusca *Ilyanassa obsolete*, *Notch* and *Su(H)* are clearly important for endoderm formation and cell fates^20^. For Annelida *H. robusta* and *C. teleta*, *Notch* participates in segment formation, as well as in vertebrates^13–15,22^. Furthermore, these genes were also shown to be associated with neurogenesis in *N. vectensis*, *S. purpuratus*, *and D. melanogaster*, but not in *P. dumerilii*^18,34,58,59^. The functional differentiation of conserved genes enriched the diversification of species. However, there are few studies on the regulatory factor *Presenilin*. Moreover, even though the expression level of some developmental stages are very low, they also performed functions, like some *Hes* genes in *D. melanogaster*^50^.

## Conclusions

In this study, we demonstrated conservation and divergence within the Notch pathway and revealed the evolution of core Notch pathway genes across metazoan genomes, with a strong focus on lophotrochozoan species. We demonstrated that the Notch pathway can be traced back to Porifera, Ctenophora and even earlier organisms, and that novel genes were differentiated in lophotrochozoans. Comparative transcriptomics revealed similarities and differences in lophotrochozoans compared to other metazoans. In addition, we identified the novel *Delta2* gene in molluscs, which may function in specific developmental stages of lophotrochozoans along with *Hes*/*Hey*-related genes, and proposed its formation mechanisms. We also discovered the expansion of target genes and the differentiation of the expression pattern of conservative genes in different species. However, future experiments are required to confirm these expression patterns and clarify gene function. Together, our study demonstrates that comparative and evolutionary analyses are essential tools for studying pathway evolution. Exploring the Notch pathway in lophotrochozoans will improve our understanding of their development and phenotype diversity, which could provide a basis for future gene function studies.

## Supplementary Information

Supplementary Data 1.

Supplementary Data 2.

Supplementary Data 3.

Supplementary Data 4.

Supplementary Data 5.

Supplementary Data 6.

Supplementary Figure 1.

Supplementary Information.

Supplementary Table 4.

Supplementary Figure 5.
