# Supplementary figures and images for: Comparative and evolutionary analyses reveal conservation and divergence of the notch pathway in lophotrochozoa
Source: Sci Rep. 2021 May 31;11:11378. doi: 10.1038/s41598-021-90800-8 (PMC8166818; doi:10.1038/s41598-021-90800-8)

a

Tree scale: 1

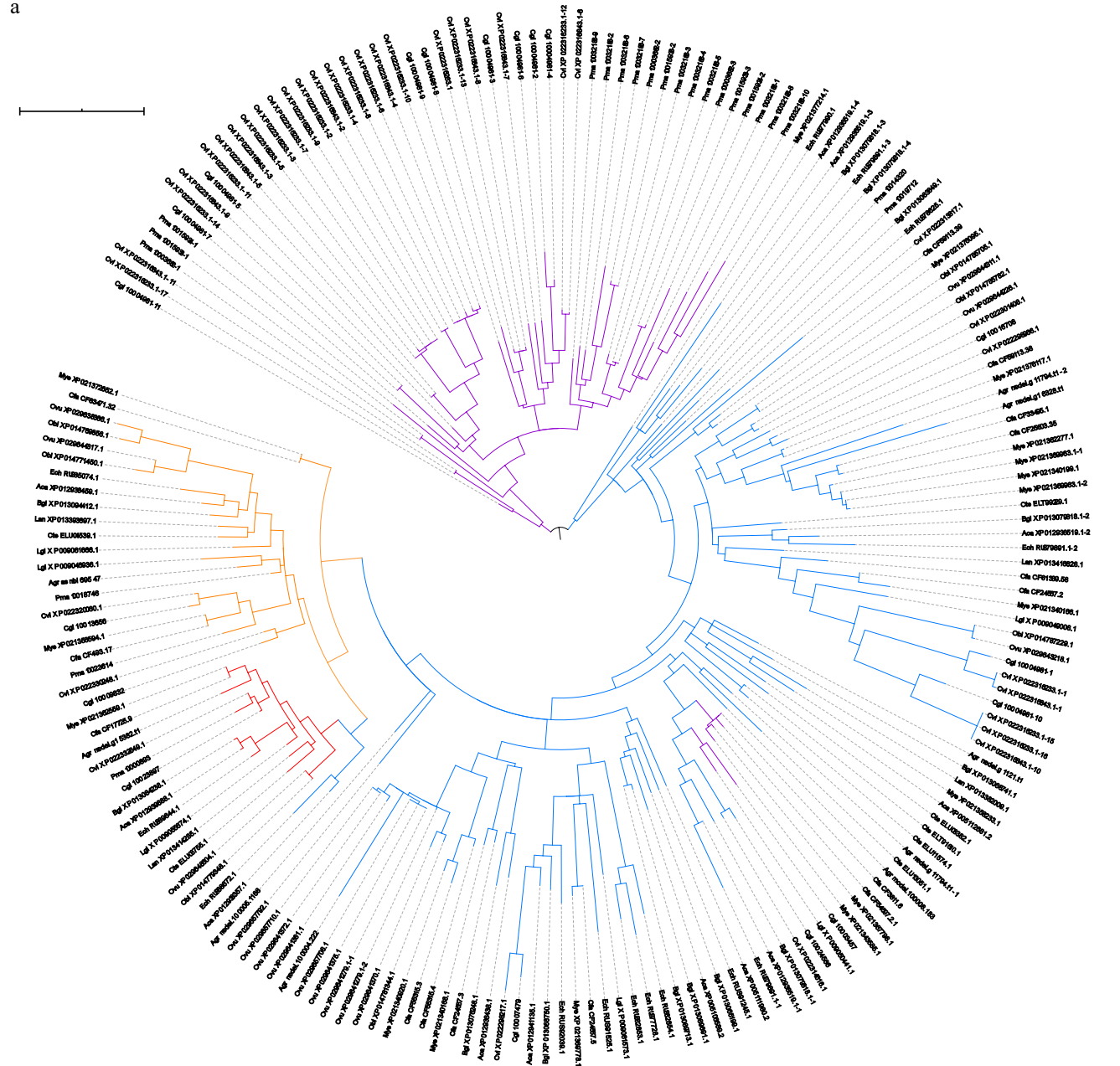

b

DSL or DSL repeat

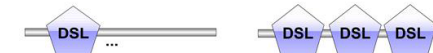

DSL+EGF

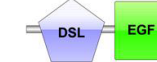

MNLL+DSL+EGF

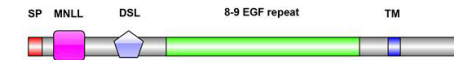

MNLL+DSL+EGF+WVC

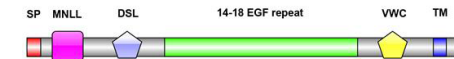

Supplement: Supplementary file 7 — Supplementary Figure 1. [file 41598_2021_90800_MOESM7_ESM.pdf]

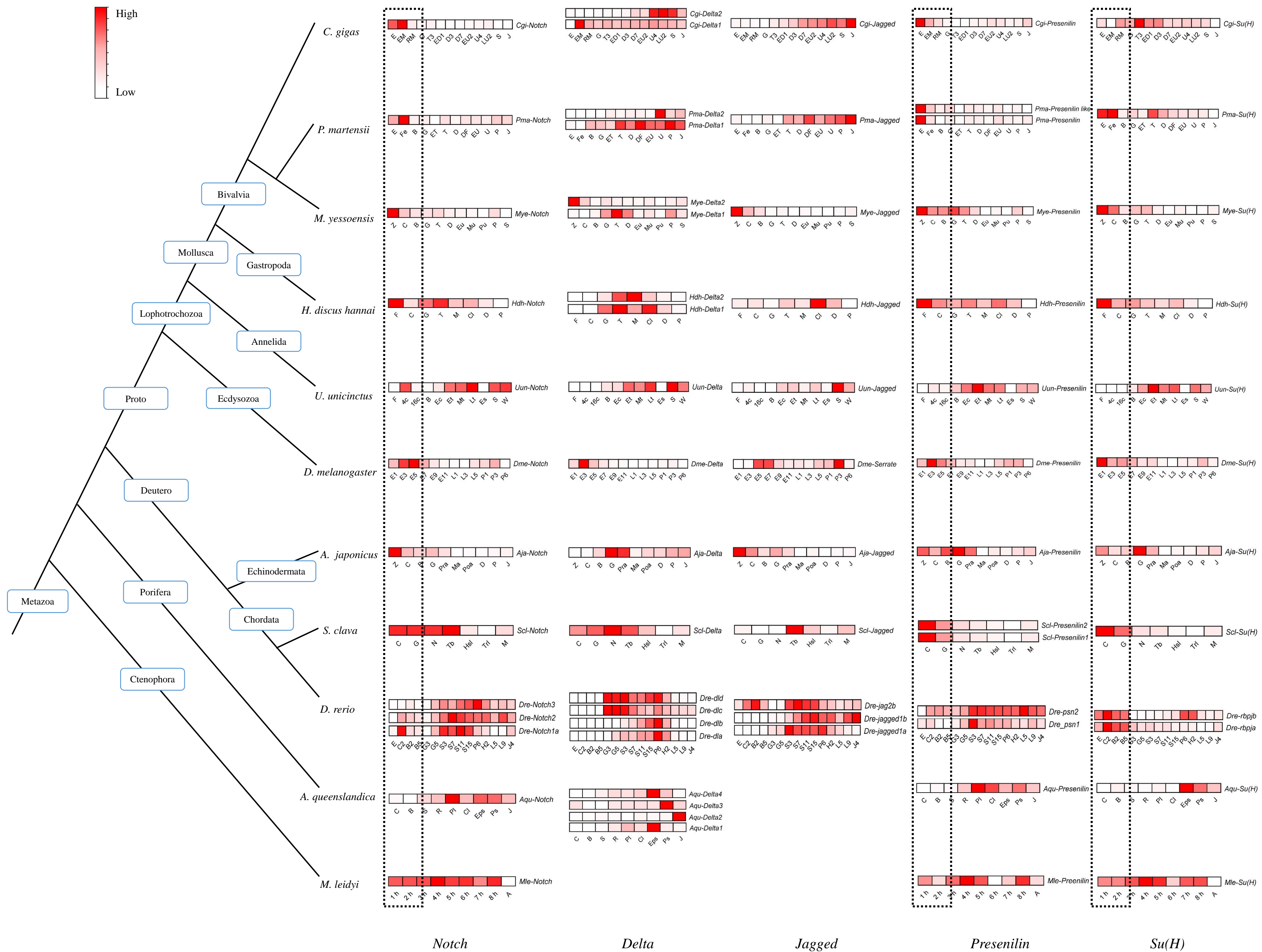

Supplement: Supplementary file 10 — Supplementary Figure 5. [file 41598_2021_90800_MOESM10_ESM.pdf]
