# Supplementary material for: Comparative and evolutionary analyses reveal conservation and divergence of the notch pathway in lophotrochozoa
Source: Sci Rep. 2021 May 31;11:11378. doi: 10.1038/s41598-021-90800-8 (PMC8166818; doi:10.1038/s41598-021-90800-8)
Supplement: Supplementary file 8 — Supplementary Information. [file 41598_2021_90800_MOESM8_ESM.pdf]

Comparative and Evolutionary Analyses Reveals the Conservation and Divergence of the Notch Pathway in the Lophotrochozoa

Xin He[a,b,c,f], **Fucun Wu**[a,b,e,f,*], **Linlin Zhang**[a,b,e,*], **Li Li**[a,d,e], **Guofan Zhang**[a,b,e]

[a] CAS and Shandong Province Key Laboratory of Experimental Marine Biology, Center for Ocean Mega-Science, Institute of Oceanology, Chinese Academy of Sciences, Qingdao, 266071, China
[b] Laboratory for Marine Biology and Biotechnology, Pilot National Laboratory for Marine Science and Technology, Qingdao 266237, China
[c] University of Chinese Academy of Sciences, Beijing 100039, China
[d] Laboratory for Marine Fisheries Science and Food Production Processes, Pilot National Laboratory for Marine Science and Technology, Qingdao 266237, China
[e] National and Local Joint Engineering Laboratory of Ecological Mariculture, Qingdao 266071, China
[f] The Innovation of Seed Design, Chinese Academy of Sciences, Wuhan 430072, China

[*] Corresponding author.
E-mail address: wufucun@qdio.ac.cn (FW), linlinzhang@qdio.ac.cn (LZ)

Table S1 Pfam Accession

Domain	**Notch**	**DSL**	**EGF**	**VWC**	**Presenilin**	**BTD**	**LAG1-DNAbind**	**HLH**
Pfam	PF00066	PF01414	PF00008	PF00093	PF01080.	PF09270.	PF09271	PF00010
Domain	**FGF**	**HH_signal**	**Insulin**	**MH2**	**Frizzled**	**Wnt**	**Activin_recp**	**TALPID3**
Pfam	PF00167	PF01085	PF00049	PF03166	PF01534	PF00110	PF01064	PF15324
Domain	**Homeobox**	**Forkhead**	**RAI16-like**	**STAT_bind**	**SH2**	**ERK-JNK_inhib**	**TGF_beta**	**Hairy_orange**
Pfam	PF00046	PF00250.	PF10257	PF02864	PF00017	PF15002	PF00019	PF07527

Table S2 The Sequence ID of the Major Genes in the Notch Pathway

	Notch	Delta	Jagged	Presenilin	Su(H)
Beroe ovata	UOYG01025213.1 UOYG01025214.1	-	-	UOYG01004404.1	UOYG01023648.1
Amphimedon queenslandica	ABZ79675.1 (NP_001266228.1)	XP_019852536.1 (*Aqu-Delta1*) XP_019858177.1 (*Aqu-Delta2*) NP_001292179.1 (*Aqu-Delta3*) NP_001292176.1 (*Aqu-Delta4*)	-	XP_019849527.1	XP_011410239.2
Nematostella vectensis	AEW42991.1 (*Nve-Notch*)	AEW42992.1 (*Nve-Delta*)	-	XP_001625236.1 (*Nve-Presenilin*)	XP_001633533.1 (*Nve-Su(H)*)
Hydra vulgaris	NP_001296638.1	XP_012561851.1	AEP83811.1	XP_012561939.1	XP_002162622.2
Hofstenia miamia	-	-	-	98014984|psn1 98020615|psn2	98031429|Su(H)
Schistosoma mansoni	XP_018647502.1 XP_018648078.1	XP_018655106.1	XP_018647863.1 XP_018649788.1 XP_018650604.1	ABS30416.1	XP_018650960.1
Echinococcus multilocularis	CDI98240.1	CDS35697.1 CDS37977.1	CDS39893.1	CDS40238.1	CDS42579.1
Lingula anatina	XP_013419105.1 (*Lan-Notch*)	XP_013393697.1 (*Lan-Delta*)	XP_013414285.1 (*Lan-Jagged*)	XP_013414065.1 (*Lan-Presenilin*)	XP_013403310.1 (*Lan-Su(H)*)
Platynereis dumerilii	CAJ38792.1	AKP06498.1	AKP06505.1	AKP06494.1	AKP06493.1
Capitella teleta	ELT93394.1	ELU04539.1	ELU03755.1	ELU08846.1	ELT98158.1
Helobdella robusta	XP_009013849.1, XP_009011066.1	XP_009015118.1	XP_009014186.1	XP_009026851.1 XP_009024256.1	XP_009017036.1 XP_009012889.1
Acanthopleura granulata	model.g31722.t1	asmbl_69547	model.g15382.t1	asmbl_61426	model.g1803.t1
Aplysia californica	XP_012943392.1	XP_012938459.1	XP_012939888.1	NP_001191418.1	XP_012944725.1
Elysia chlorotica	RUS91728.1	RUS85074.1	RUS89644	RUS69332.1	RUS90631.1
Biomphalaria	XP_013093464.1	XP_013094412.1	XP_013084238.1	XP_013065482.1	XP_013090963.1

glabrata					
Lottia gigantea	XP_009044486	XP_009061666.1<br>(*Lgi-Delta1*)<br>XP_009046936.1<br>(*Lgi-Delta2*)	XP_009055674.1	XP_009047025.1	ESO97471.1
Haliotis discus hannai	T07122<br>(*Hdh-Notch*)	T19567<br>(*Hdh-Delta1*)<br>T26181<br>(*Hdh-Delta2*)	T18809<br>(*Hdh-Jagged*)	T19349<br>(*Hdh-Presenilin*)	T13741<br>(*Hdh-Su(H)*)
Crassostrea gigas	CGI_10013186<br>(XP_011450291.1)<br>(*Cgi-Notch*)	CGI_10009832<br>(*Cgi-Delta1*)<br>CGI_10013656<br>(*Cgi-Delta2*)	CGI_10023897<br>(*Cgi-Jagged*)	CGI_10019579<br>(*Cgi-Presenilin*)	CGI_10018383<br>(*Cgi-Su(H)*)
Crassostrea virginica	XP_022322171.1	XP_022330248.1<br>XP_022320060.1	XP_022332849.1	XP_022332245.1	XP_022335897.1
Mizuhopecten yessoensis	XP_021352656.1	XP_021372652.1<br>XP_021356594.1	XP_021362559.1	XP_021349855.1	OWF47943.1
Chlamys farreri	CF64469.3	CF63471.32<br>CF493.17	CF17725.9	CF22817.10	CF64623.4
Pinctada fucata martensii	Pma_10025422<br>(*Pma-Notch*)	Pma_10018746<br>(*Pma-Delta1*)<br>Pma_10023614<br>(*Pma-Delta2*)	Pma_10000893<br>(*Pma-Jagged*)	Pma_10014008<br>(*Pma-presenilin*)<br>Pma_10009132<br>(*Presenilin-like*)	Pma_10008559<br>(*Pma-Su(H)*)
Octopus bimaculoides	XP_014776889.1	XP_014769856.1<br>XP_014771450.1	XP_014779348.1	XP_014785182.1	XP_014779920.1
Octopus vulgaris	XP_029649022.1	XP_029638366.1<br>XP_029644317.1	XP_029648604.1	XP_029642727.1	XP_029650905.1
Caenorhabditis elegans	NP_499007.1<br>NP_499014.1	NP_001024615.1	-	NP_508175.1,<br>NP_492095.1	NP_001293739.1
Drosophila melanogaster	NP_001245510.1<br>(*Dme-Notch*)	NP_477264.1<br>(*Dme-Delta*)	NP_524527.3<br>(*Dme-Serrate*)	AAB53369.1<br>(*Dme-Presenilin*)	NP_476868.1<br>(*Dme-Su(H)*)
Apis mellifera	XP_006568051.2	XP_006566475.1	XP_006564793.1	XP_396620.5	XP_016767749.1
Strongylocentrotus purpuratus	XP_797451.4	NP_001027542.1	XP_011678556.1<br>XP_011678630.1<br>XP_011675859.1	XP_001178715.1	XP_011673426.1
Apostichopus japonicus	PIK60374.1<br>(*Aja-Notch*)	PIK54747.1<br>(*Aja-Delta*)	PIK49767.1<br>(*Aja-Jagged*)	PIK44116.1<br>(*Aja-Presenilin*)	PIK43190.1<br>(*Aja-Su(H)*)
Saccoglossus kowalevskii	XP_006818779.1	NP_001158418.1	XP_002740519.2	XP_002738746.1	XP_006811939.1
Styela clava	XP_039270515.1<br>(*Scl- Notch*)	XP_039248161.1<br>(*Scl-Delta*)	XP_039254307.1<br>(*Scl -Jagged*)	XP_039260250.1<br>(*Scl-Presenilin1*)<br>XP_039254919.1<br>(*Scl-Presenilin2*)	XP_039265104.1<br>(*Scl-Su(H)*)
Branchiostoma	XP_019643529.1	XP_019627312.1	XP_019633701.1	XP_019630833.1	XP_019626796.1

belcheri					XP_019647425.1
Danio rerio	NP_571516.1 (*Dre-Notch1a*) NP_571377.2 (*Dre-Notch1b*) NP_001108566.2 (*Dre-Notch2*) NP_571624.2 (*Dre-Notch3*)	NP_571029.2 (*Dre-dla*) NP_571033.1 (*Dre-dlb*) NP_571019.1 (*Dre-dlc*) NP_571030.2 (*Dre-dld*)	XP_005168261.1 (*Dre-jag1a*) NP_571938.2 (*Dre-jag1b*) XP_005160542.1 (*Dre-jag2b*)	NP_571099.1 (*Dre-psn1*) NP_571589.2 (*Dre-psn2*)	NP_942579.2 (*Dre-rbpja*) NP_001077020.1 (*Dre-rbpjb*)
Mus musculus	Notch1-NP_032740.3 Notch2-NP_035058.2 Notch3-NP_032742.1 Notch4-NP_035059.2	XP_011244569.1 NP_031892.2 NP_062327.2	NP_038850.1 XP_011242300.1	NP_001349200.1 NP_001122077.1	NP_001074397.1 NP_033062.1
Homo sapiens	Notch1-NP_060087.3 Notch2-NP_077719.2 Notch3-NP_000426.2 Notch4-NP_004548.3	NP_005609.3 NP_061947.1 NP_058637.1	NP_002217.3 NP_000205.1	NP_000012.1 NP_000438.2	NP_001268377.1 NP_001350506.1

Table S3 The *Hes*/*Hey*-related Genes among metazoans

	Hes	Hey	Helt	Cwo
Beroe ovata	-	-	-	-
Amphimedon queenslandica	-	AquHey-XP_003387453.1	-	-
Nematostella vectensis	NveHes2-AEW42994.1 or XP_001635328.1 NveHes4-AEW42997.1 NveHes3-XP_001635326.1	NveHey-AEW42993.1	-	-
Hydra vulgaris	HvuHes-XP_004208005.1	-	-	-
Hofstenia miamia	HmiHes-98048776	HmiHey-98033833	-	-
Schistosoma mansoni	SmaHes1-XP_018647563.1 SmaHes2-XP_018653208.1	SmaHey1-XP_018649397.1 SmaHey2-XP_018649396.1	-	-
Echinococcus	EmuHes-CDS38030.1	EmuHey-CDS426	-	-

multilocularis		97.1		
Lingula anatina	LanHES1-XP_013381587<br>LanHES2-XP_013381582<br>LanHES3-XP_013383390<br>LanHES4-XP_013383388<br>LanHES5-XP_013403236<br>LanHES6-XP_013412582<br>LanHES7-XP_013412583<br>LanHES8-XP_013412580<br>LanHES9-XP_013395724<br>LanHES10-XP_013395728<br>LanHES11-XP_013395690<br>LanHES12-XP_013390924	LanHey-XP_0134<br>14701	-	LanCwo1-XP_013419897<br>LanCwo2-XP_013380691
Platynereis<br>dumerilii	PduHes1-AGS55435.1<br>PduHes2-AGS55436.1<br>PduHes3-AGS55437.1<br>PduHes4-AGS55438.1<br>PduHes5-AGS55439.1<br>PduHes6-AGS55440.1<br>PduHes7-AGS55441.1<br>PduHes8-AGS55442.1<br>PduHes9-AGS55443.1<br>PduHes10-AGS55444.1<br>PduHes11-AGS55445.1<br>PduHes12-AGS55446.1<br>PduHes13-AGS55447.1	PduHey1-AGS554<br>48.1	-	PduCwo-AGS55449.1
Capitella teleta	CteHes1-ELU08377<br>CteHes2-ELU08376<br>CteHes3-ELU10814<br>CteHes4-ELU10815<br>CteHes5-ELT92193	CteHey1-ELU089<br>81<br>CteHey2-ELU089<br>80	-	CteCwo-ELU02556
Helobdella<br>robusta	HroHES1-XP_009020819<br>HroHES2-XP_009022784<br>HroHES3-XP_009023789<br>HroHES4-XP_009023790<br>HroHES5-XP_009023792	HroHey-XP_0090<br>14197	-	-
Acanthopleura<br>granulata	AgrHes1-model.g30019.t1<br>AgrHes2-model.g30018.t1<br>AgrHes3-model.g30014.t1<br>AgrHes4-model.g30046.t1<br>AgrHes5-model.g30020.t1<br>AgrHes6-model.g31256.t1<br>AgrHes7-model.g29508.t1	AgrHey-model.g1<br>9192.t1	AgrHelt-model.g4531.t1	-
Aplysia	AcaHes1-XP_005109821	AcaHey-XP_0129	--	AcaCwo-XP_012935276

californica	AcaHes2-XP_012945161<br>AcaHes3-XP_005104468	40775		
Elysia chlorotica	EchHes1-RUS81509.1<br>EchHes2-RUS85462.1<br>EchHes3-RUS85031.1<br>EchHes4-RUS87118.1<br>EchHes5-RUS83438.1<br>EchHes6-RUS72576.1	EchHey-RUS8730<br>0.1	-	EchCwo-RUS91494.1
Biomphalaria<br>glabrata	BglHes1-XP_013095166<br>BglHes2-XP_013088757<br>BglHes3-XP_013075104<br>BglHes4-XP_013087343<br>BglHes5-XP_013088081	BglHey-XP_01306<br>7329	-	BglCwo-XP_013080510
Lottia gigantea	LgiHes1-XP_009064533<br>LgiHes2-XP_009058075<br>LgiHes3-XP_009057811<br>LgiHes4-XP_009058863<br>LgiHes5-XP_009049759<br>LgiHes6-XP_009053615<br>LgiHes7-XP_009053062<br>LgiHes8-XP_009058074<br>LgiHes9-XP_009064532<br>LgiHes10-XP_009064534<br>LgiHes11-XP_009064535	LgiHey-XP_00904<br>6688	LgiHelt-XP_009045357	LgiCwo-XP_009053558
Haliotis discus<br>hannai	HdhHes1-T00679<br>HdhHes2-T00683	HdhHey-T01691	-	HdhCwo-T09094
Crassostrea gigas	CgiHes1-XP_011426791<br>CgiHes2-XP_011426827<br>CgiHes3-XP_011434420<br>CgiHes4-XP_011442866<br>CgiHes5-XP_011434419<br>CgiHes6-XP_011456332<br>CgiHes7-XP_011456334<br>CgiHes8-XP_011456335<br>CgiHes9-XP_011418427<br>CgiHes10-XP_011418430	CgiHey-XP_01144<br>6705	CgiHelt-XP_011454244	CgiCwo1-XP_011423628<br>CgiCwo2-XP_011423478
Crassostrea<br>virginica	CviHes1-LOC111132480<br>CviHes2-LOC111133867<br>CviHes3-LOC111099184<br>CviHes4-LOC111135582<br>CviHes5-LOC111132221<br>CviHes6-LOC111132222<br>CviHes7-LOC111138134	CviHey-LOC1111<br>31773	CviHelt-LOC111120884	CviCwo1-XP_022302549.1<br>CviCwo2-XP_022301976.1
Mizuhopecten	MyeHes1-XP_021342518	MyeHey-XP_0213	MyeHelt-XP_021340917	MyeCwo-XP_021371008

yessoensis	MyeHes2-XP_021364693<br>MyeHes3-XP_021350063<br>MyeHes4-XP_021372905<br>MyeHes5-XP_021363795<br>MyeHes6-XP_021355435	56185		
Chlamys farreri	CfaHes1-CF20445.6<br>CfaHes2-CF57753.3<br>CfaHes3-CF59079.38<br>CfaHes4-CF64955.1<br>CfaHes5-CF59079.39	CfaHey-CF63447.3	CfaHelt-CF55315.5	CfaCwo-CF43523.5
Pinctada fucata martensii	PmaHes1-10022586<br>PmaHes2-10012161<br>PmaHes3-10022584	PmaHey1-10000662<br>PmaHey2-10019999	PmaHelt-10020900	PmaCwo1-10029931
Octopus vulgaris	OvuHes-XP_029657891.1	-	OvuHelt-XP_029638240.1	OvuCwo-XP_029635006.1
Octopus bimaculoides	ObiHes-XP_014772623	ObiHey-XP_014767679	ObiHelt-XP_014781134	ObiCwo-XP_014773539
Caenorhabditis elegans	CelHes-NP_500281.1	-	-	-
Drosophila melanogaster	DmeHes1-NP_524509 (*Dme-E(spl)m3*)<br>DmeHes2-NP_524505 (*Dme-E(spl)mbeta*)<br>DmeHes3-NP_536753 (*Dme-E(spl)m7*)<br>DmeHes4-NP_524504 (*Dme-E(spl)mgamma*)<br>DmeHes5-NP_525094 (*Dme-Hesr*),<br>DmeHes6-HLH-NP_524503 (*Dme-E(spl)mdelta*)<br>DmeHes7-NP_524511 (*Dme-E(spl)m5*)<br>DmeHes8-NP_524513 (*Dme-E(spl)m8*)<br>DmeHes9-NP_476923 (*Dme-dpn*),<br>DmeHes10-NP_523599 (*Dme-Sidpn*)<br>DmeHes11-NP_523977 (*Dme-hairy*)	DmeHey-NP_523657 (*Dme-Hey*)	-	DmeCwo-NP_524775 (*Dme-Cwo*)
Apis mellifera	AmeHes1-XP_393312.1<br>AmeHes2-XP_001120327.2<br>AmeHes3-XP_003249471.2<br>AmeHes4-XP_001120814.2<br>AmeHes5-XP_006559646.1	AmeHey-XP_026299131.1	-	AmeCwo-XP_026300267.1

	AmeHes6-XP_393948.3			
Strongylocentrotus purpuratus	SpuHes1-NP_001001768.2<br>SpuHes2-XP_787040.2<br>SpuHes3-XP_796692.2<br>SpuHes4-XP_030843279.1<br>SpuHes5-XP_030843275.1	SpuHey-XP_783676.2	-	-
Apostichopus japonicus	AjaHes1-PIK61026.1<br>AjaHes2-PIK35797.1<br>AjaHes3-PIK37883.1<br>AjaHes4-PIK58850.1	AjaHey-PIK40047.1	-	-
Saccoglossus kowalevskii	SkoHes1-NP_001158466.1	SkoHey1-NP_001158467.1<br>SkoHey2-XP_006811443	SkoHelt-NP_001161564.1	
Styela clava	SclHes1-XP_039251338.1<br>SclHes2-XP_039259018.1<br>SclHes3-XP_039250505.1	SclHey-XP_039261038.1	-	-
Branchiostoma belcheri	BbeHes1-XP_019637861.1<br>BbeHes2-XP_019637894.1<br>BbeHes3-XP_019637880.1<br>BbeHes4-XP_019628064.1<br>BbeHes5-XP_019628076.1<br>BbeHes6-XP_019628086.1<br>BbeHes7-XP_019628069.1<br>BbeHes8-XP_019633323.1<br>BbeHes9-XP_019627214.1<br>BbeHes10-XP_019633783.1	BbeHey1-XP_019641636.1<br>BbeHey2-XP_019647772.1	-	-
Danio rerio	DreHes1-NP_571155.1 (*Dre-her3*)<br>DreHes2-NP_571154.2 (*Dre-her6*)<br>DreHes3-NP_571948.1 (*Dre-her9*)<br>DreHes4-NP_001116717.1 (*Dre-hes2.1*)<br>DreHes5-NP_001038818.1 (*Dre-hes2.2*)<br>DreHes6-NP_955918.3 (*Dre-her8a*)<br>DreHes7-XP_003199574.1 (*Dre-her15.1*)<br>DreHes8-XP_021324225.1 (*Dre-E(spl)m7*)<br>DreHes9-NP_878295.1 (*Dre-her15.1*)<br>DreHes10-NP_001159638.1 (*Dre-her8.2*)	DreHey1-NP_997726.1 (*Dre-hey1*)<br>DreHey2-NP_571697.2 (*Dre-hey2*)<br>DreHey3-NP_859425.1 (*Dre-heyl*)	DreHelt-NP_996948.1 (*Dre-helts*)	-

Mus musculus	MmuHes1-NP_032261.1<br>MmuHes2-NP_001288734.1<br>MmuHes3-NP_032263.2<br>MmuHes4-NP_001357684.1<br>MmuHes5-NP_062352.1<br>MmuHes6- NP_149030.2	MmuHey1-NP_03<br>4553.2<br>MmuHey2-NP_03<br>8932.1<br>MmuHey3-NP_03<br>8933.2	MmuHelt-NP_776150.2	-
Homo sapiens	HsaHes1-NP_001019769<br>HsaHes2-NP_001010926<br>HsaHes3-NP_061962<br>HsaHes4-NP_061115<br>HsaHes5-NP_005515<br>HsaHes6-NP_066993<br>HsaHes7-NP_003661<br>HsaHes8-NP_110389	HsaHey1-NP_036<br>390<br>HsaHey-2NP_036<br>391<br>HsaHey-3NP_055<br>386	HsaHelt-NP_001025058	-

Table S4 The FPKM/RPKM and abbreviations of the developmental stages.
See excel file “supplementary Table S4.xlsx”.

Table S5 The Core Domains of Developmental Pathways.

	TGF-β[a]			Wnt[b]		Jak/STAT[c]		RPTK[d]		Notch[e]			Hedgehog[f]		RA[g]	Fox[h]	Hox[i]	ERK[j]
	Activin_recp	MH2	TGF_beta	Wnt	Frizzled	STAT_bind	SH2	Insulin	FGF	**EGF**	**DSL**	**bHLH**	HH_signal	TALPID3	RAI16-like	Forkhead	Homeobox	ERK-JNK_inhib
Bov	2	6	8	5	4	3	29	-	5	57	25	43	-	-	-	22	69	1
Aqu	2	4	8	5	5	2	87	-	2	225	12	23	1	-	2	19	33	1
Nve	3	5	7	19	7	1	25	2	14	643	24	51	2	-	2	27	94	1
Hvu	2	4	12	11	3	1	47	1	12	172	16	29	4	2	2	16	46	1
Hmi	1	6	6	6	8	1	60	-	3	33	1	39	1	1	-	17	90	2
Sma	1	9	2	8	9	-	33	-	-	43	6	35	1	-	2	16	75	1
Emu	-	7	3	7	9	-	44	-	-	42	8	31	2	-	2	16	81	-
Lan	4	10	11	13	5	2	65	2	3	1427	6	77	1	2	2	27	114	2
Cte	3	4	14	12	5	5	42	2	1	571	7	82	1	3	2	42	145	1
Hro	2	9	8	19	4	2	60	-	-	230	3	67	1	-	1	32	243	1
Agr	2	5	12	10	5	2	51	4	5	227	11	67	1	2	2	35	144	1
Ech	2	3	13	12	5	1	46	6	3	138	13	64	2	1	1	21	93	1
Aca	1	5	12	16	5	1	52	4	4	292	12	64	2	2	2	19	90	1
Bgl	1	7	14	18	5	2	55	2	3	280	16	73	1	2	3	18	86	1
Lgi	4	5	10	12	5	2	28	3	2	334	6	77	1	1	3	31	141	1
Hdh	3	4	9	14	6	1	58	3	4	447	19	77	6	1	2	25	145	-
Cgi	4	8	13	13	5	1	51	2	1	633	19	78	1	2	2	26	131	1
Cvi	4	13	18	13	5	3	78	4	2	821	24	101	3	2	2	33	144	1
Mye	4	6	13	12	5	3	63	2	2	593	27	69	6	2	2	25	134	1
Cfa	3	6	14	13	6	3	55	2	2	450	16	66	6	1	2	27	130	1
Pma	2	6	12	10	8	2	52	1	2	475	10	59	3	2	2	39	134	-
Ovu	1	5	10	12	7	1	50	1	2	425	16	51	2	-	2	16	82	1
Obi	1	6	11	13	5	1	42	1	4	267	7	48	3	1	2	19	81	1

Cel	3	20	5	5	8	3	43	1	2	88	12	54	-	-	1	24	131	1
Dme	5	6	7	8	6	1	31	6	3	105	2	55	1	-	1	18	108	-
Ame	10	5	8	6	2	1	35	2	4	139	2	71	1	-	2	16	99	1
Spu	2	8	18	16	8	1	60	5	5	2933	21	65	1	2	2	24	130	1
Aja	1	5	13	16	5	1	71	1	-	661	2	35	-	3	2	24	82	1
Sko	2	7	23	13	6	1	45	2	7	1161	6	63	1	2	3	41	142	1
Scl	3	4	14	17	8	3	55	-	6	452	3	46	2	2	2	35	115	2
Bbe	5	5	16	13	7	2	62	5	10	1499	2	86	3	2	2	27	141	1
Dre	13	15	14	26	15	8	147	14	32	417	10	149	4	1	8	69	232	1
Mmu	11	8	41	20	12	7	116	11	22	278	4	138	4	1	5	43	305	1
Hsa	10	7	21	40	12	8	128	15	26	336	4	126	4	1	4	48	290	1
Total	117	233	435	453	220	76	1966	104	193	**16894**	**372**	**2259**	72	41	74	937	4300	33

The line means none. The domains of Notch Pathway are underlined.

a Activin is a TGF receptor; MH2 is a domain of Smad; TGF_beta is a multifunctional peptide of TGF-β.

b Wnt is a family of Wnt signaling pathway. Frizzled is related to Wnt and Hedgehog.

c STAT proteins are a family of transcription factors of Jak/STAT, SH2 is a conserved domain included in both STAT and RPTK signaling pathway.

d Insulin, FGF and EGF are subfamilies of receptor protein-tyrosine kinase (RPTK) pathway.

e EGF is contained in the Notch ligand as well. DSL is the key domain of Notch ligands while bHLH is the key domain of Notch target genes.

f HH_signal is an amino-terminal signaling domain of Hedgehog pathway. TALPID3 is a family of eukaryotic proteins that are targets for Hedgehog signaling;

g RAI16-like is a retinoic acid-induced protein 16-like proteins of retinoic acid (RA) signaling pathway.

h Forkhead domain is a conserved DNA-binding domain of fox gene.

i Homebox is a domain of transcriptional regulators which encoded by Hox genes.

j ERK-JNK_inhib is an inhibitor of extracellular signal-regulated kinases (ERK).

Colors of background for the species represent different phylum. White is Ctenophora, light grey is Porifera, light blue is Cnidaria, dark blue is Xenacoelomorpha, blue is Lophotrochozoa, orange is Ecdysozoa, red is Ambulacrum (Echinodermata and Hemichordata) and green is Chordata (the same below). *Platynereis dumerilii* was discarded due to lack of proteome.

Abbreviation of species are showed in Materials & Methods.

Table S6 Domains of the Major Genes in the Notch Pathway

	Notch	DSL	EGF	VWC	Presenilin	BTD	LAG1-DN Abind	bHLH	Hairy orange
Bov	8	25	57	9	1	1	1	43	7
Aqu	5	12	225	4	1	1	1	23	1
Nve	3	24	643	10	1	1	1	51	5
Hvu	5	16	172	12	1	1	1	29	-
Hmi	-*	1*	33*	3*	1	1	1	39	1
Sma	2	6	43	-	1	1	1	35	1
Emu	4	8	42	1	1	1	1	31	-
Lan	13	6	1427	52	1	1	1	77	10
Cte	7	7	571	17	1	1	1	82	8
Hro	5	3	230	5	2	2	2	67	2
Agr	6	11	227	52	1	1	1	67	11
Ech	3	13	138	26	1	1	1	64	7
Aca	6	12	292	39	1	1	1	64	5
Bgl	8	16	280	16	1	1	1	73	10
Lgi	5	6	334	41	1	1	1	77	7
Hdh	5	19	447	30	1	2	2	77	11
Cgi	6	19	633	44	1	1	1	78	10
Cvi	7	24	821	74	1	1	1	101	9
Mye	8	27	593	42	1	1	1	69	7
Cfa	8	16	450	32	1	1	1	66	7
Pma	7	10	475	38	3	1	1	59	6
Ovu	8	16	425	38	1	1	1	51	1
Obi	5	7	267	39	1	1	1	48	4
Cel	6	12	88	12	2	1	1	54	-
Dme	4	2	105	9	1	1	1	55	11
Ame	4	2	139	34	1	1	1	71	9
Spu	6	21	2933	91	1	1	1	65	7
Aja	4	2	661	60	1	1	1	35	4
Sko	8	6	1161	45	1	1	1	63	5
Scl	5	3	452	47	2	2	2	46	3
Bbe	6	2	1499	90	1	2	2	86	11
Dre	16	10	417	68	2	3	3	149	15
Mmu	17	4	278	60	2	2	2	138	11
Hsa	18	4	336	63	2	2	2	126	11
Total	228	371	16861	1200	42	42	42	2259	217

Figure S1. DSL domain phylogeny across lophotrochozoans. (a) The phylogenetic tree was constructed using the DSL domains from 138 DSL family genes using maximum likelihood methods. Clades with different colours represent different domain arrangements shown in (b). Red indicates *Jagged* containing DSL, EGF, MNLL, and VWC. Orange indicates *Delta* containing DSL, EGF, and MNLL. Blue represents genes containing DSL and EGF domains. Purple represents genes containing DSL or DSL repeats but not EGF. Different DSL domains in the same gene are indicated by '-' after the gene ID. (b) Domain diagram of DSL family. DSL domain protein sequences are given in Supplementary Data 2.

See "Figure S1.pdf" file.

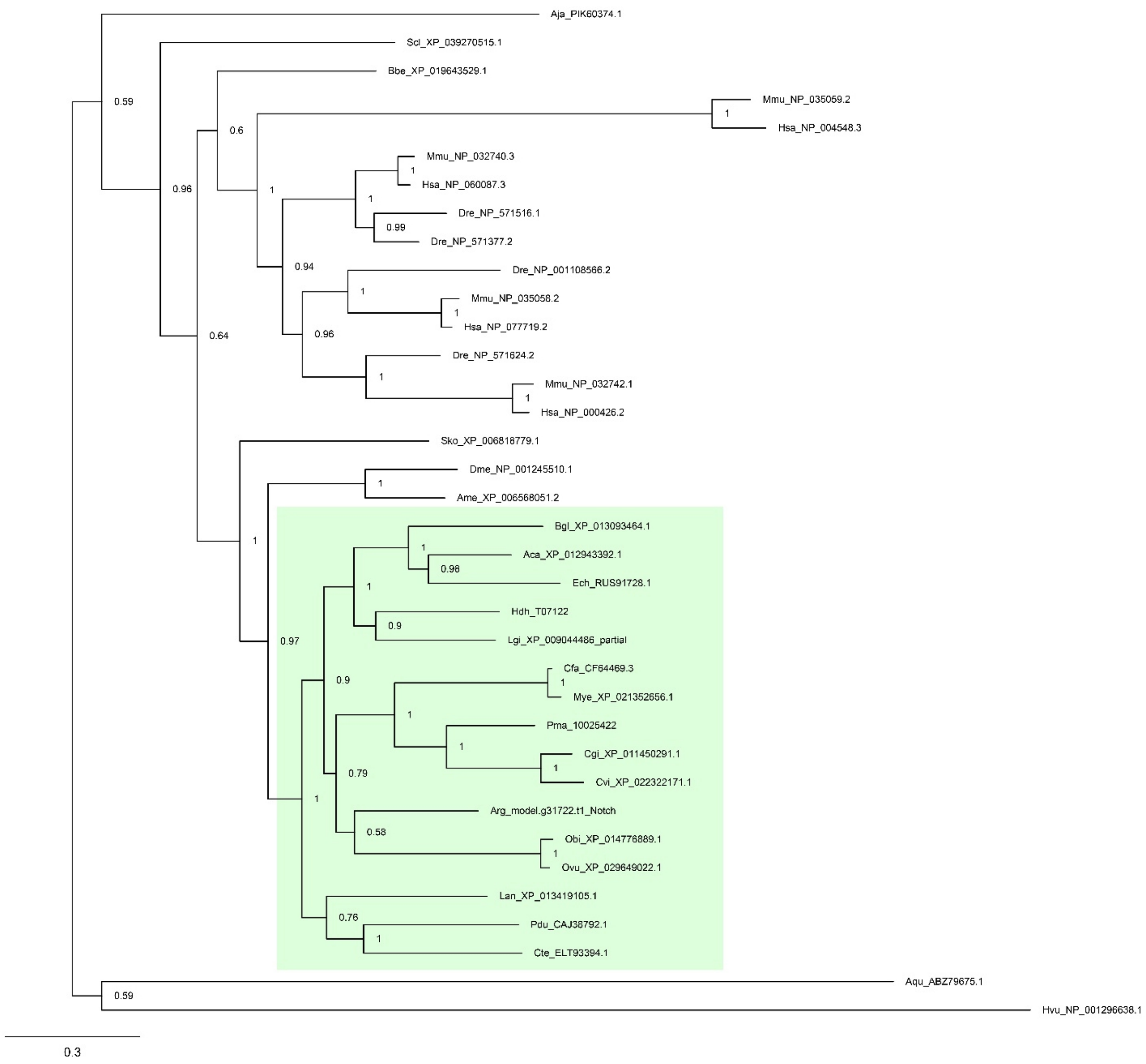

Figure S2. Phylogeny of *Notch* genes across metazoans. The phylogenetic tree was constructed using 36 *Notch* genes from 27 complete metazoan genomes using the maximum likelihood method. Fasttree support values are shown at the basal node. Lophotrochozoans are shown in green. The protein sequences of these genes are shown in Supplementary Data 4.

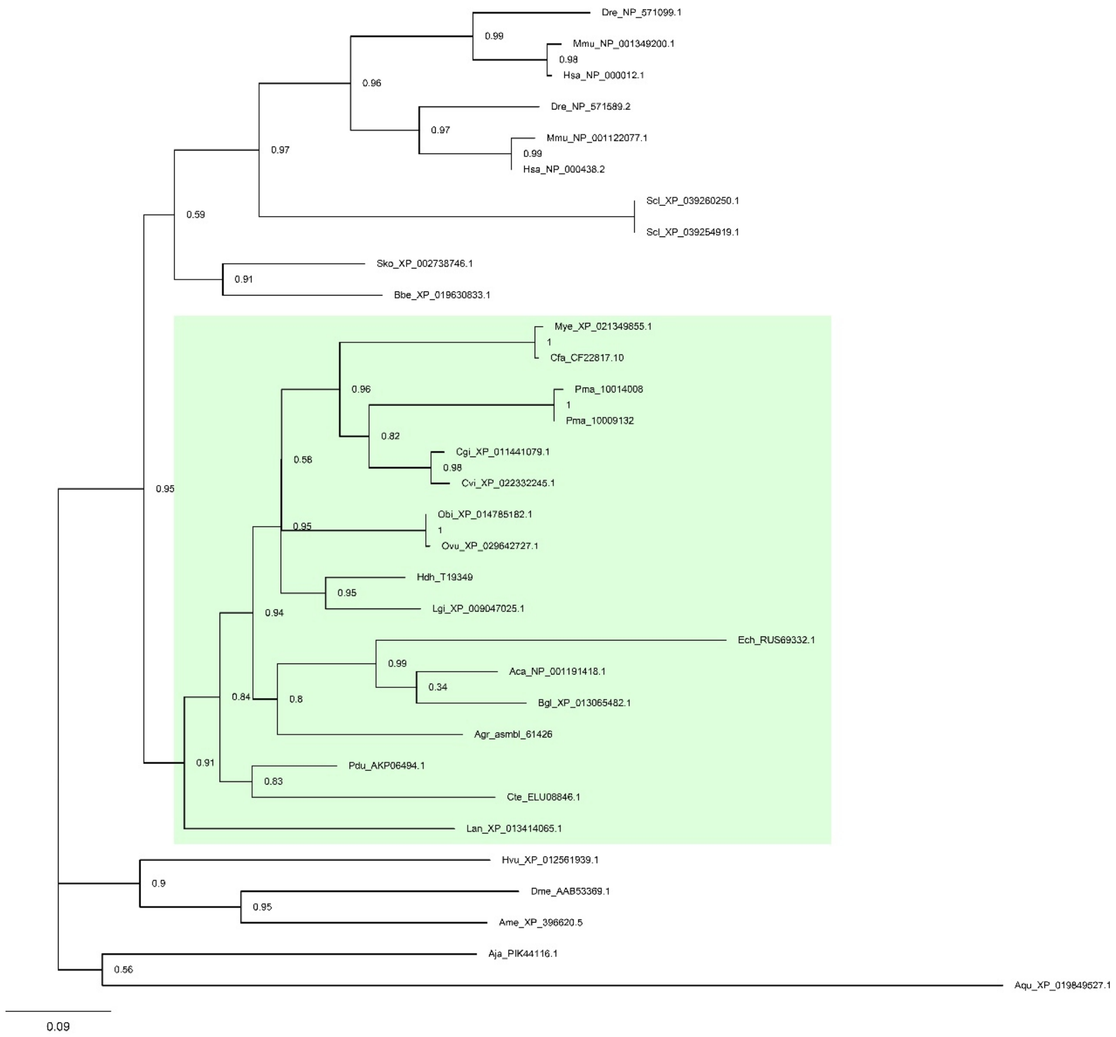

Figure S3. Phylogeny of *Presenilin* genes across metazoans. The phylogenetic tree was constructed using 32 *Presenilin* genes from 27 complete metazoan genomes using the maximum likelihood method. Fasttree support values are shown at the basal node. Lophotrochozoans are shown in green. The protein sequences of these genes are shown in Supplementary Data 5.

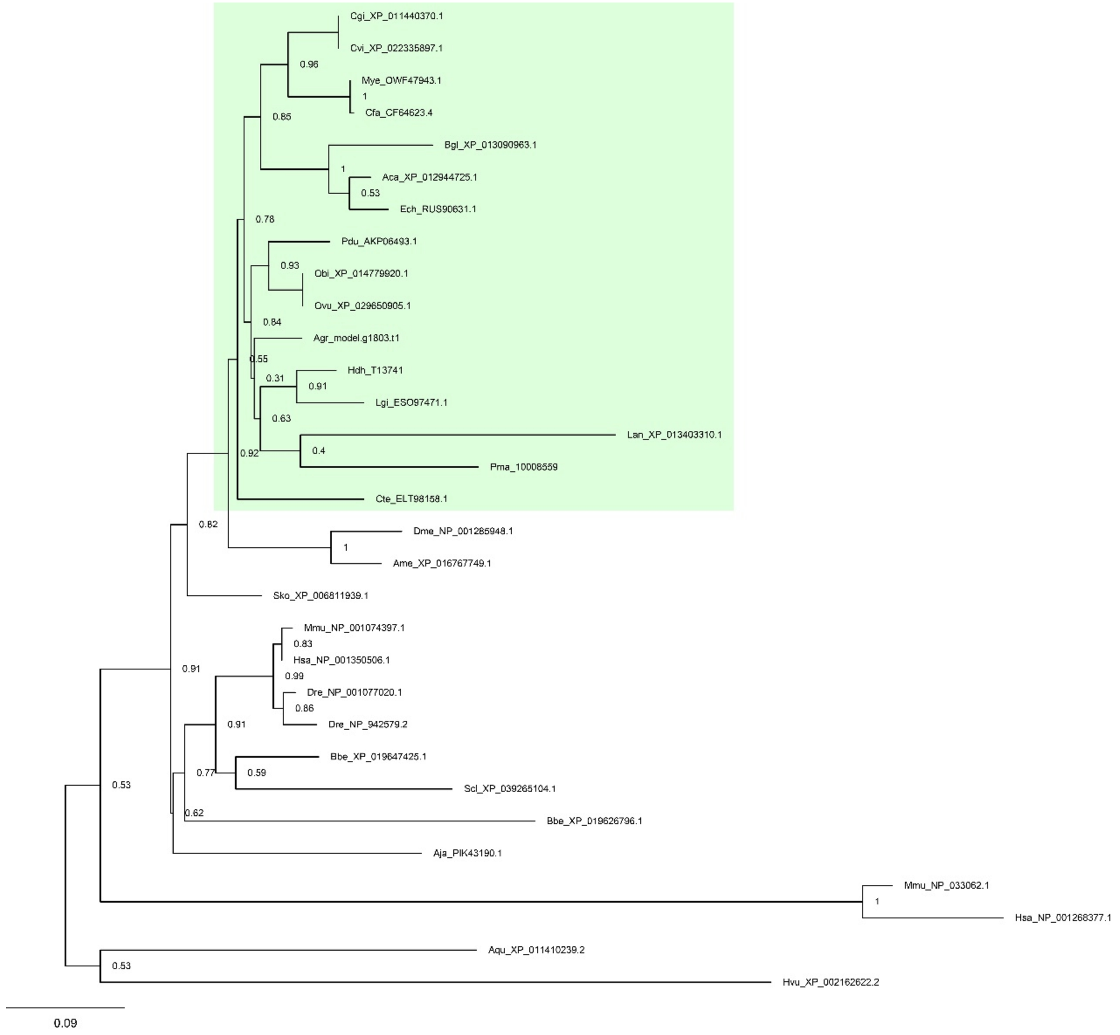

Figure S4. Phylogeny of *Su(H)* genes across metazoans. The phylogenetic tree was constructed using 31 *Su(H)* genes from 27 complete metazoan genomes using the maximum likelihood method. Fasttree support values are shown at the basal node. Lophotrochozoans are shown in green. The protein sequences of these genes are shown in Supplementary Data 6.

Figure S5. Transcriptomic data of the Notch pathway genes (*Notch*, *Delta*, *Jagged*, *Presenilin,* and *Su(H)*) in eleven species throughout development. The heatmaps show the expression trends. Red means high while white means low. Sequence IDs are shown in Supplementary Table S2 and S3. Developmental stage abbreviations are described in Supplementary Table S4.

See “Figure S5.pdf” file.
